# No Association Between Functional Polymorphisms in COMT and MTHFR and Schizophrenia Risk in Korean Population

**DOI:** 10.4178/epih/e2010011

**Published:** 2010-12-24

**Authors:** Ho Jin Kang, Byeong Moo Choe, Seong Hwan Kim, Seung-Rak Son, Kyoung-Mu Lee, Byoung Gwon Kim, Young-Seoub Hong

**Affiliations:** 1 Department of Preventive Medicine, Dong-A University School of Medicine, Busan, Korea.; 2 Department of Psychiatry, Dong-A University School of Medicine, Busan, Korea.; 3 Department of Environmental Health, Korea Open University, Seoul, Korea.

**Keywords:** Catechol-O-methyltransferase, Schizophrenia, Methylenetetrahydrofolate reductase, Polymorphism

## Abstract

**OBJECTIVES:**

Common genetic SNPs in two genes, encoding catechol-O-methyltransferase (COMT) and methylenetetrahydrofolate reductase (MTHFR), which are interconnected with COMT gene regulation, have been reported to contribute to schizophrenia risk. In this study, we evaluated the association between functional polymorphisms in COMT and MTHFR and schizophrenia risk with a case-control study in a Korean population.

**METHODS:**

We performed a case-control study by genotyping analysis using 360 cases and 348 controls in Korean subjects to determine the association between functional polymorphisms in COMT and MTHFR and schizophrenia risk.

**RESULTS:**

Four functional SNPs in COMT (Val158Met and rs165599) and MTHFR (C677T and A1298C) were genotyped by primer extension assay. None of the genotype distributions for the four SNPs was significantly different between cases and controls. Stratified analysis did not show any significant gender difference for any polymorphism. In addition, we found no evidence of a gene-gene interaction in the analysis of combined genotypes.

**CONCLUSION:**

Our results suggest no significant association between the selected functional polymorphisms of COMT or MTHFR in Korean schizophrenia subjects. However, further studies are required to confirm our findings in a larger number of subjects.

## INTRODUCTION

Schizophrenia is a complex disorder involving multiple genetic and environmental factors [1]. Despite accumulating evidence suggesting that the etiology of schizophrenia includes a highly heritable component [2], the specific genetic loci and neurobiological mechanisms that contribute to the disorder remain unclear.

A number of studies have evaluated the association between the Val108/158Met polymorphism (rs4680) in the COMT gene, encoding catechol-O-methyltransferase, which catalyzes the metabolism of dopamine, and schizophrenia risk. Although several functional studies have shown that patients with the Val/Val genotype had better executive function and working memory compared to Met allele carriers [3, 4], recent metaanalyses could not replicate the association in either European or Asian populations [5-7]. Another variant in the 3' untranslated region (rs165599) in the COMT gene, which was highly associated with schizophrenia in a large study conducted in a population of Israelis of Ashkenazi descent [8], has been reported to differentially affect the expression of Val108/158Met alleles in human brain tissue [9].

The MTHFR gene, encoding methylenetetrahydrofolate reductase, which plays an important role in folate metabolism [10], is known to have two single nucleotide polymorphisms (SNPs; C677T and A1298C) affecting enzyme activity [11, 12]. The MTHFR 677T variant showed a 35% reduction in MTHFR activity such as DNA methylation [13, 14]. A recent meta-analysis supports the association between MTHFR C677T and A1298C SNPs and the development of schizophrenia [15-18].

The metabolic pathways of COMT and MTHFR are interconnected [19]. Genetic variation in MTHFR could influence the expression of COMT function through DNA methylation of the COMT promoter region [20]. COMT promoter methylation deficits have been described in schizophrenia, with concordant increases in COMT expression [21]. It has been suggested that abnormal methylation contributes to executive dysfunction in schizophrenia and other psychiatric disorders through downstream effects on dopamine signaling [22].

In the present study, we examined the relationship between functional polymorphisms in COMT and MTHFR polymorphisms and schizophrenia risk. We also investigated the effect of the combined genotypes of COMT and MTHFR polymorphisms.

## SUBJECTS AND METHODS

### Study subjects

A total of 360 patients with chronic schizophrenia and 348 healthy controls matched by age and sex were recruited from the inpatient and outpatient clinical services of the Department of Psychiatry, Dong-A University Hospital and other university-affiliated hospitals of Busan City Psychiatric Center. Schizophrenia was diagnosed on the basis of a clinical interview, using the Comprehensive Assessment of Symptoms and History (CASH) [23]. All patients met the DSM-IV criteria for schizophrenia [24]. The DSM-IV-diagnosed schizophrenic patients were recruited to the study between Jan 2003 and Dec 2009 from the inpatient population. Patients were selected according to strict criteria and those with both a current and past history of aggressive behavior. Healthy adult Korean volunteers were recruited as controls from among the residents of Busan when receiving health checkups. They had no reported current or past history of psychiatric disorders, including significant violent episodes. The demographic data for the case-control study is as follows: 54% male cases and 56% male controls (p=0.948), with a mean age of 38.32±3.93 yr for the cases and 38.26±5.51 for the controls (p=0.866). This study design was approved by the Committee on Human Research of the Dong-A University Hospital (Dong-A University IRB 07-18) and written informed consent was obtained from all participants or their legal guardians.

### DNA extraction and COMT/MTHFR genotyping

Blood samples were collected in tubes that contained EDTA. Genomic DNA was extracted from white blood cell fractions by means of a Qiagen blood kit (Qiagen, Chatsworth, CA, USA). All four SNPs selected in this study were genotyped by SNP-IT™ assays using the SNPstream 25K™ System, which has been customized to perform fully automated genotyping using samples in 384-well plates with a colorimetric readout (Orchid Biosciences, New Jersey, USA).

Briefly, for each SNP, a set of three primers was designed: two PCR primers were selected to amplify a 100-200 base pair product under standard conditions and a single-base primer extension (SBE) primer was designed to be approximately 25 bp in length on one side of the SNP site. Automated liquid handling robotics was used to set up 5 µL PCR reactions in 384-well plates. Each PCR reaction contained: 10.0 ng of DNA, 1X PCR buffer, 0.125 units of AmpliTaq Gold DNA polymerase (ABI, USA), 3.0 mM MgCl_2_, 0.25 mM of each dNTP, and 0.5 pmol of each primer. Reactions were incubated at 95℃ for 10 min, then cycled 35 times at (95℃ for 30 s, 50, 55 or 60℃ for 1 min, 72℃ for 1 min) followed by 72℃ for 5 min. An annealing temperature of 50, 55 or 60℃ was chosen as appropriate. The primer sequences are shown in Table 1.

The amplified PCR products were digested with T7 exonuclease (0.45 U/µL) at room temperature for 30 min. The 5' phosphothioates on one of the PCR primers protected one strand of the PCR product from T7 exonuclease digestion, resulting in the generation of a single-stranded PCR product. The single-stranded PCR product was hybridized to a 384-well plate that contained a covalently attached SNP-IT™ primer extension primer designed to hybridize immediately adjacent to the SNP. After hybridization, the SNP-IT™ primer was extended for a single base with the Klenow fragment of DNA polymerase I and a mixture of appropriately labeled terminating nucleotides, which were labeled with either FITC or biotin and were complementary to the SNP. The identity of the incorporated nucleotide was determined using serial colorimetric reactions with anti-FITC-AP (alkaline phosphatase) and streptavidin-HRP (horseradish peroxidase) using p-nitrophenyl phosphate (PNPP) and tetramethylbenzidine (TMB) as a substrate respectively.

The results of yellow and/or blue color developments for each sample (wells) were analyzed in an ELISA reader and the final genotype calls were automatically assigned using the QCReview™ program (Orchid Biosciences). Automated genotype calls were corroborated by visual inspection of the data.

### Statistical analyses

The chi-square test was used to identify departures from the Hardy-Weinberg equilibrium among the controls. To estimate the relative risk of schizophrenia in relation to SNP genotype, odds ratios (ORs) and 95% confidence intervals (CIs) were calculated using unconditional logistic regression. The homozygote of the most common allele in the subjects was used as the reference and a p value <0.05 was considered statistically significant.

Stratified analyses were also conducted to evaluate whether the association between the SNPs and schizophrenia risk was derived from the different ages and genders. All statistical analyses were performed using SAS version 9.1 (SAS Inc., Cary, NC, USA).

## RESULTS

The distribution of age and gender was similar between cases and controls. The mean age was 38.3±3.9 in the cases, and 38.3±5.5 in the controls. The proportion of male subjects was 55% and 56% for cases and controls, respectively (data not shown). All genotype frequencies of the four SNPs, COMT Val158Met (rs4680), rs165599 and MTHFR C677T (rs1801133), and A1298C (rs1801131), were consistent with the Hardy-Weinberg equilibrium in the control group (p>0.05). As shown in Table 2, the genotype frequencies of the COMT Val158Met and rs165599 did not differ between the patients and controls. Likewise, the genotype frequencies of the MTHFR C677T and A1298C did not differ between the patients and controls.

No association was found either in the stratified analyses by gender-difference (Table 3), or in the analyses of combined genotypes (Table 4). In addition, we found no evidence of an interactive effect between COMT Val158Met and MTHFR C677T in the patients and controls (Table 5).

## DISCUSSION

In our study, no association was found between the four functional SNPs in COMT and MTHFR, and schizophrenia risk in a Korean population.

Although a number of previous studies have evaluated the association between SNPs in the COMT gene, particularly a well-known polymorphism at COMT Val158Met termed rs4680, and schizophrenia, the results have been inconsistent and recent meta-analyses found no evidence for a relationship between COMT and schizophrenia [5-7, 25]. Several reports suggest an association with the highly active Val allele [3, 26], but many other studies have found no significant association [6, 27-29]. In our results, the COMT Val158Met genotype frequency was founded by 53.9% for GG (Val), 38.6% for GA (Val/Met) and 7.5% for AA (Met) in schizophrenia. These frequencies did not differ significantly with control subjects (55.2% for GG, 37.9% for GA and 6.9% for AA). This results were similar with that of previous studies conducted on Korean populations (schizophrenia: 61% for GG, 31% for GA and 7% for AA; Control: 52% for GG, 39% for GA and 9% for AA) [30]. And the no significant association for this SNP with schizophrenia was similar among several Asian subjects but very different among several European subjects [6, 31-33]. Overall, the Val allele may be a small risk factor for schizophrenia in European populations, while case-control studies have shown no association between Val158Met and schizophrenia in Asian populations, and thus the results remain inconclusive.

Another candidate polymorphism in the 3'-flanking region (rs165599) was not associated with schizophrenia risk in our study. The 3'-flanking region of SNP rs165599 exhibited significant allelic differences in expression in the human brain, and the A allele in schizophrenia patients is associated with higher expression of the COMT gene [9]. A previous study by Chien et al. [34] reported that rs165599 was associated with a family study, but there was no significant association in a case-control study. Okochi et al. [25] reported that there was no association between four major functional SNPs (rs2075507, rs737865, rs6267 and rs165599) and schizophrenia in a Japanese population. Therefore, these results indicate that the Val/Met polymorphism and three other functional SNPs may not play a major role in schizophrenia.

In addition, Roffman et al. [35] reported that there was contributed to the lack of association between Val158Met and rs165599 and schizophrenia, which may have gender-specific differences, as there is a stronger association of the G (Val) allele with schizophrenia in females [8]. Meyer-Lindenberg et al. found that the Val158Met genotype involved an interaction between rs2097603 at the P2 promoter region and rs165599 in the 3'-flanking region. These interactions have been implicated in an inefficient prefrontal working memory response during a working memory paradigm in healthy control subjects [36]. In our analysis of the combination of COMT Val158-Met and rs165599, we found no interactive effect in the patients and control subjects.

A number of studies including a meta-analysis have suggested an association between schizophrenia and MTHFR C677T, and A1298C and schizophrenia [15-17]. Zintzaras reported that the C677T association was only of marginal significance in an East Asian population, with a fixed effects odds ratio of 1.23 (1.00-1.52), whereas for Caucasians the results were non-significant [18]. However, there was no association between two MTHFR functional SNPs and schizophrenia risk in our study. Such inconsistent results may be due to the ethnic diversity in terms of allele frequency and exposure to environmental factors, warranting further studies.

Finally, we also carried out an association analysis on the effect of two genetic variants of enzymes of the methylation pathway, the COMT Val158Met and MTHFR C677T polymorphisms, on schizophrenia risk. MTHFR C677T may contribute to COMT gene regulation, wherein T-allele carriers exhibit diminished promoter methylation, increased COMT expression, and reduced dopamine signaling [22]. Roffman et al. reported that the MTHFR T allele undermines category generation in schizophrenia, with 33% of T/T patients unable to complete a single category, and then reported that COMT Val108/158Met does not significantly influence this aspect of executive function, by itself or in interaction with MTHFR C677T [35, 37]. However, that study had several limitations such as the number of subjects, especially for a gene interaction study, and the results require replication in other samples. Our study revealed that no association between COMT 158Val genotype combined with the MTHFR 677TT within patient and controls. Therefore, the genetic interaction of the COMT Val158Met and MTHFR C677T genotype may contribute to executive function deficits in schizophrenia [37].

The limitations of our study include the small sample size, the lack of sub-classification of schizophrenia, and the lack of adjustment for potential confounders. Our study had a power of >70% in each gene to detect an odds ratio of ≥1.5 (assuming a dominant effect, with a minor allele frequency of 0.1 and α=0.05). However, we note that further studies are required on a larger number of subjects and compressive approach for the COMT and MTHFR genes in the Korean population.

In conclusion, our results suggest no significant association between any of the selected functional polymorphisms of COMT or MTHFR in Korean schizophrenia subjects. However, further studies are required to confirm our findings in a larger number of subjects.

## Figures and Tables

**Table 1:**
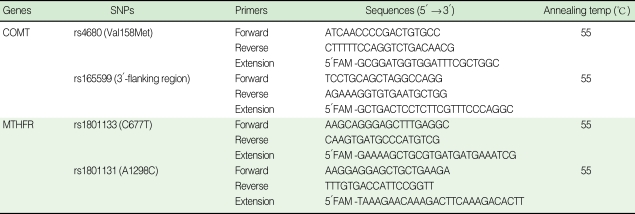
Primer sequences for genotyping

COMT, catechol-O-methyltransferase; MTHFR, methylenetetrahydrofolate reductase.

**Table 2:**
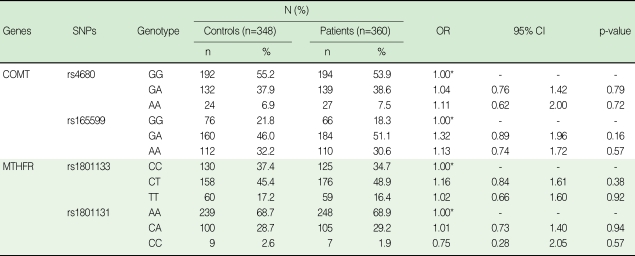
Genotype frequencies of selected polymorphisms in COMT and MTHFR among cases and controls

OR, odds ratio; CI, confidence interval; COMT, catechol-O-methyltransferase; MTHFR, methylenetetrahydrofolate reductase. ^*^Reference category, OR=1.

**Table 3:**
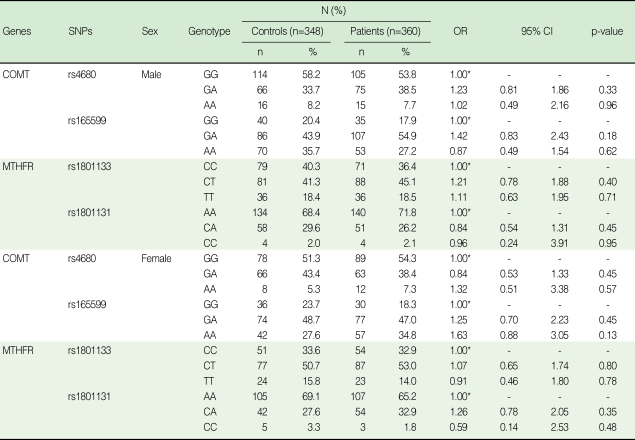
Stratified analysis by gender for the association between selected polymorphisms in COMT and MTHFR and schizophrenia risk

OR, odds ratio; CI, confidence interval; COMT, catechol-O-methyltransferase; MTHFR, methylenetetrahydrofolate reductase. ^*^Reference category, OR=1.

**Table 4:**
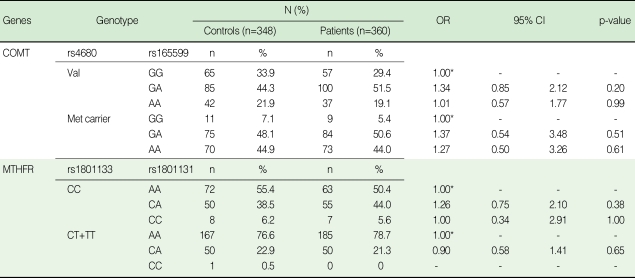
Distribution of combined genotypes of COMT Val158Met, rs165599 and MTHFR C677T, A1298C among cases and controls

OR, odds ratio; CI, confidence interval; COMT, catechol-O-methyltransferase; MTHFR, methylenetetrahydrofolate reductase. ^*^Reference category, OR=1, Val genotype; GG, Met carrier genotype; GA+AA.

**Table 5:**
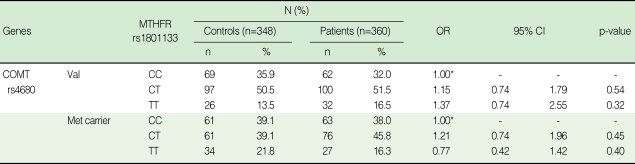
Interactive effects between COMT Val158Met and MTHFR C677T on schizophrenia risk

OR, odds ratio; CI, confidence interval; COMT, catechol-O-methyltransferase; MTHFR, methylenetetrahydrofolate reductase. ^*^Reference category, OR=1, Val genotype; GG, Met carrier genotype; GA+AA.
